# Differentiating Primary Tumors for Brain Metastasis with Integrated Radiomics from Multiple Imaging Modalities

**DOI:** 10.1155/2022/5147085

**Published:** 2022-09-26

**Authors:** Guoquan Cao, Ji Zhang, Xiyao Lei, Bing Yu, Yao Ai, Zhenhua Zhang, Congying Xie, Xiance Jin

**Affiliations:** ^1^Radiology Department, The First Affiliated Hospital of Wenzhou Medical University, Wenzhou, China 325000; ^2^Radiotherapy Center, The First Affiliated Hospital of Wenzhou Medical University, Wenzhou, China 325000; ^3^Medical and Radiation Oncology, The Second Affiliated Hospital of Wenzhou Medical University, Wenzhou, China 325000; ^4^School of Basic Medical Science, Wenzhou Medical University, Wenzhou, China 325000

## Abstract

**Objectives:**

To differentiate the primary site of brain metastases (BMs) is of high clinical value for the successful management of patients with BM. The purpose of this study is to investigate a combined radiomics model with computer tomography (CT) and magnetic resonance imaging (MRI) images in differentiating BMs originated from lung and breast cancer.

**Methods:**

Pretreatment cerebral contrast enhanced CT and T1-weighted MRI images of 78 patients with 179 BMs from primary lung and breast cancer were retrospectively analyzed. Radiomic features were extracted from contoured BM lesions and selected using the Mann–Whitney *U* test and the least absolute shrinkage and selection operator (LASSO) logistic regression. Binary logistic regression (BLR) and support vector machine (SVM) models were built and evaluated based on selected radiomic features from CT alone, MRI alone, and combined images to differentiate BMs originated from lung and breast cancer.

**Results:**

A total of 10 and 6 optimal radiomic features were screened out of 1288 CT and 1197 MRI features, respectively. The mean area under the curves (AUCs) of the BLR and SVM models using fivefolds cross-validation were 0.703 vs. 0.751, 0.718 vs. 0.754, and 0.781 vs. 0.803 in the training dataset and 0.708 vs. 0.763, 0.715 vs. 0.717, and 0.771 vs. 0.805 in the testing dataset for models with CT alone, MRI alone, and combined CT and MRI radiomic features, respectively.

**Conclusions:**

Radiomics model based on combined CT and MRI features is feasible and accurate in the differentiation of the primary site of BMs from lung and breast cancer.

## 1. Introduction

With the improvements in cancer management over the past few decades, the survival of cancer patients has been increased [1]. This leads to an increasing incidence and detection of brain metastases (BMs) with the availability of more precise and innovative neuroimaging modalities [2, 3]. BM has become the most common intracranial tumor and an important cause of morbidity and mortality in adults, which occurs in up to 30% of adult cancer patients [4]. Occasionally, BMs are detected before diagnosing their primary tumor sites. Previous studies demonstrated that about 2–15% of patients with BMs do not have an existing cancer diagnosis and their primary cancer cannot be identified despite investigations [5].

The differentiation of the primary sites is of high clinical importance for the successful management of patients with BMs, as the treatment and prognosis of BMs are highly dependent on the molecular characteristics of the primary tumors [6], especially for targeted therapies and immunotherapy [7, 8]. With the emergence of radiomics, extracting quantitative features from clinical imaging arrays has become a promising noninvasive differentiation method for BMs [9]. Kniep et al. demonstrated an area under curve (AUC) of 0.64 and 0.82 in the prediction of BMs from non-small-cell lung cancer (NSCLC) and melanoma using magnetic resonance imaging (MRI) radiomics, respectively [10]. Ortiz-Ramón et al. also used MRI images to classify BMs by their primary sites using radiomic approach and achieved AUCs of 0.963 ± 0.054, 0.936 ± 0.070, and 0.607 ± 0.180 in the differentiating BMs from lung vs. breast cancer, lung cancer vs. melanoma, and breast cancer vs. melanoma, respectively [11, 12].

MRI is the preferred modality for detecting brain lesions and differentiation, but with mixed differentiation accuracy for BMs. Computer tomography (CT) is also an accepted primary modality for BMs screening, and it is extremely useful for screening new neurological signs or symptoms with or without a history of malignancy [13]. In previous studies, only the random forest was applied for model construction with single image modality. The purpose of this study is to investigate the feasibility and accuracy of a combined radiomics analysis with multiple images of MRI and CT in the differentiating BMs originated from primary lung cancer and breast cancer.

## 2. Materials and Methods

### 2.1. Patients and Images

By searching the electronic medical records, patients with confirmed BMs originated from lung cancer and breast cancer treated in the authors' institute between January 2016 and June 2020 were retrospectively reviewed and analyzed. The study was conducted in accordance with the Declaration of Helsinki and approved by the Ethics Committee in Clinical Research (ECCR) of the authors' hospital (ECCR#2019059). The need of written informed consent was waived with confirmation of patient data confidentiality by ECCR for this retrospective study.

All the enrolled patients had pretreatment cerebral CECT and contrast-enhanced T1-weighted MRI images. Cerebral CT images were acquired by a CT simulator with a 16-detector row (Brilliance, Phillips) under identical scanning parameters: 100 kV, 180–280 mA at 3 mm slice thickness. BM patients were immobilized with a thermal plastic in supine position for radiotherapy simulation with 100 mL iodinated contrast material injected intravenously at a rate of 3.0 to 4.0 mL/s via a high-pressure injector before CT scans. T1-weighted MRI was performed using a 1.5 T (MAGNETOM Avanto, Erlangen, German) or 3.0 T MRI scanner (Philips Achieva 3.0 T, Ohio, United States) with intravenously injected iodinated contrast material before MRI scans. CT images were not normalized with a pixel spacing resampled to 1 × 1 × 1 mm^3^ as they were acquired by the same CT simulator. MRI images were normalized with scale equal to 100 because they were acquired by two different MRI scanners. The pixel spacing of MRI image was resampled to 2 × 2 × 2 mm^3^ in this study.

### 2.2. Feature Extraction

**Figure 1:**
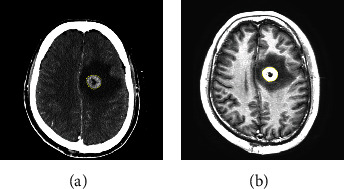
Typical contours of brain metastases on CT and MRI images.

Brain tumors in the CT and MRI images were manually contoured by a same junior radiation oncologist and verified by a senior radiation oncologist through 3D Slicer software (version 4.2.1, https://www.slicer.org) (CT images) and ITK-SNAP (version 3.6.0, https://www.itksnap.org) (MRI), respectively. For patients with 2 or more metastases, all tumors and all slices of tumor were contoured. Radiomic features were extracted and analyzed for individual BM lesion. Typical contours in CECT and MRI are shown in Figure 1.

Radiomic features were extracted from contoured regions of interest (ROIs) from the CT and MRI using the Python package (PyRadiomics) [14]. Extracted features comprised of first-order features, shape features, and texture features, such as grey-level co-occurrence matrix (GLCM), grey-level run-length matrix (GLRLM), grey-level size-zone matrix (GLSZM), and grey-level dependence matrix (GLDM) features. Based on the adding sigma levels Laplacian of Gaussian (Log) filters and Wavelet filters, a total of 1288 features and 1197 features were extracted from each lesion in CT and MRI, respectively, according to the scoring/selection criteria of PyRadiomics (http://pyradiomics.readthedocs.io/). The extraction parameters for the radiomic features of CT and MRI images are shown in Supplementary Material.

### 2.3. Feature Selection and Model Building

The enrolled patients were randomly divided into a training dataset (70%) and a testing dataset (30%) according to individual tumors. In the training dataset, potential informative radiomic features were selected firstly by the Mann–Whitney *U* tests for those with a *P* < 0.05. Then, the “elastic net” was used to select the optimal features by combining the least absolute shrinkage and selection operator (LASSO) and the Ridge Regression [15]. A tenfold cross-validation was applied to avoid overfitting, and the parameter of the elastic net (*λ*) was tuned to maximize the AUC of receiver operating characteristic (ROC) curve to select key features.

Logistic regression models and support vector machine (SVM) models were built and evaluated based on selected radiomic features from CT alone, MRI alone, and combined images. The performance of the radiomic models was assessed by the mean AUCs of fivefold cross-validation in the training dataset and independently validated on the testing dataset.

### 2.4. Statistical Analysis

The Mann–Whitney *U* test was performed with SPSS software (version 19.0, IBM, Armonk, NY, USA). The LASSO logistic regression and logistic regression model were performed using R analysis platform (version 3.0.1) along with the “glmnet” package (http://www.Rproject.org). The SVM model was achieved by the “e1071” package. The ROC curve was performed by python module (scikit-learn). For all tests, *P* < 0.05 was considered as statically significant.

## 3. Results

### 3.1. Patients' Characteristics

**Figure 2:**
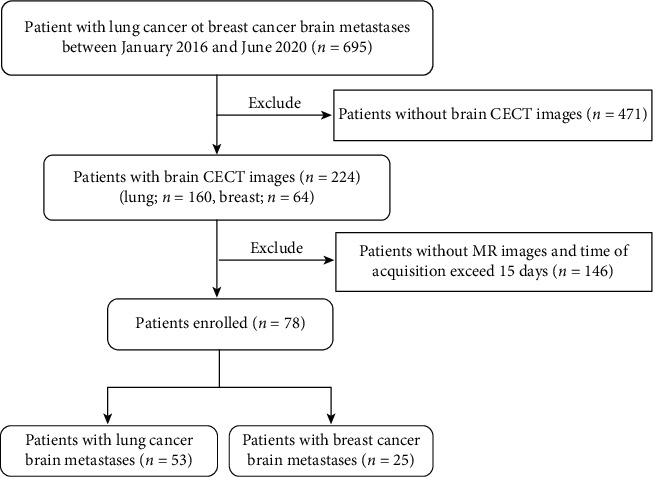
Flowchart of patient selection for this study.

The flowchart for patient selection is shown in Figure 2. A total of 78 patients with BM treated from January 2016 to June 2020 with primary lung and breast cancer were reviewed, in which 53 (38 males, 15 females) patients originated from lung cancer with a total of 95 BMs with a median age of 62 years old (range from 46 to 79) and 25 patients (1 male, 24 females) originated from breast cancer with a total of 84 BMs with a median age of 52 years old (range from 33 to 77).  Table 1 shows the demographic and clinical characteristics of the enrolled patients according to individual tumors. The clinical characteristics of the patients according to individual patient are shown in Table s1 of the Supplementary Material.

### 3.2. Selected Features

**Figure 3:**
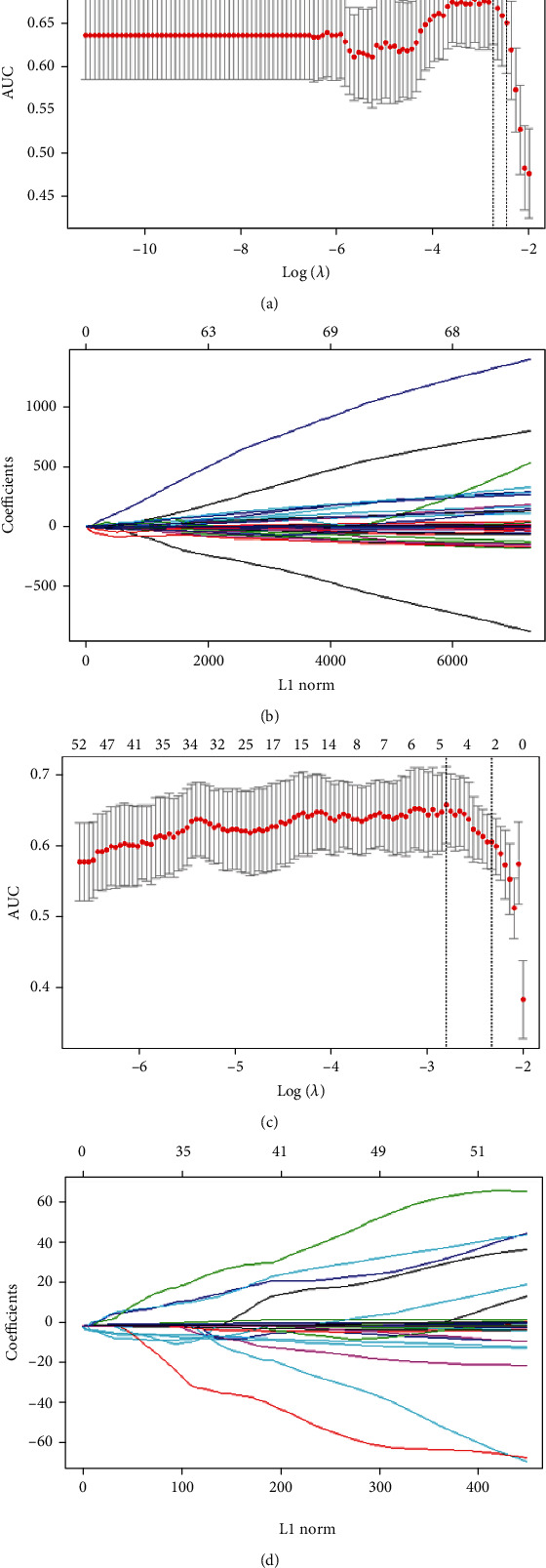
Optimal radiomic features screening using the elastic net method (a) and (c) tuning parameter (*λ*) in the elastic net using tenfold cross-validation via maximum area under curve and criterion of minimum standard deviation (b) and (d) the coefficient profiles of selected radiomic features against the L1 norm (inverse proportional to log (*λ*). (a) and (b) for CT images and (c) and (d) for MRI images.

There were 116 and 132 features selected from 1288 and 1197 features and correlated with primary site in the CECT images and MRI, respectively, according to the Mann–Whitney *U* test with a *P* < 0.05. As shown in Figure 3, a total of 10 and 6 optimal radiomic features were screened out of 116 CT features and 132 MRI features, respectively, based on the LASSO logistic model and the tune of *λ*. The selected 10 features from CT included 2 first-order features, 3 GLCM features, 3 GLSZM features, and 2 GLRLM features. The selected 6 features from MRI included 2 first-order features, 1 GLCM features, and 3 GLSZM features, as shown in Table 2.

### 3.3. Performance of Models

**Figure 4:**
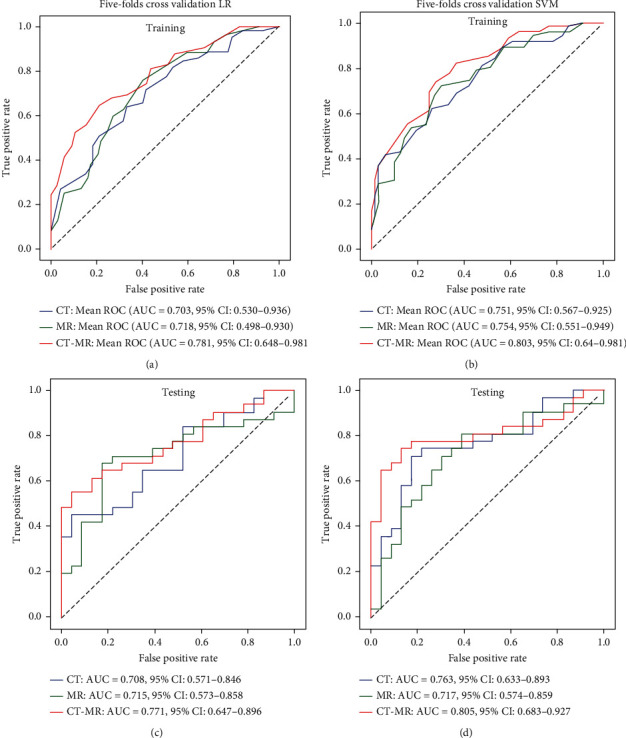
Models evaluation with mean receiver operation characteristic curves and values of area under curves for CT radiomic features alone, MRI radiomic features alone, and combined CT and MRI radiomic features in the training (a, c) and testing dataset (b, d).

Three logistics regression models and three SVM models were built according to features extracted from CT alone, MRI alone, and combined CT and MRI, respectively. In the training dataset, the achieved mean AUCs with fivefold cross-validation of the three logistic regression models and three SVM models in the differentiation BMs from lung and breast cancer were 0.703 vs. 0.751, 0.718 vs. 0.754, and 0.781 vs. 0.803 for CT alone, MRI alone, and combined CT and MRI, respectively. In the testing dataset, the achieved AUCs of the three logistic regression models and three SVM models in the differentiation BMs from lung and breast cancer were 0.708 vs. 0.763, 0.715 vs. 0.717, and 0.771 vs. 0.805 for CT alone, MRI alone, and combined CT and MRI, respectively. The ROC curves of these six models are shown in Figure 4. A detailed comparison among these six models in the testing dataset is presented in Table 3.

## 4. Discussion

In this study, the feasibility and accuracy of differentiating the primary lung and breast cancer for patients with BM based on combined radiomic features extracted from brain CECT and MRI were investigated. A best AUC of 0.805 was achieved for models built on combined CT and MRI radiomic features in the differentiation between BMs originated from lung and breast cancer.

BM is the most common type of brain tumors in adults with relatively poor prognosis [16, 17]. It was reported that over 70% BM patients have multiple brain lesions at the time of diagnosis [18]. In this study, the average number of brain metastasis was 2.3 for the enrolled patients, with an average number of 1.8 and 3.4 BMs for patients originated from lung cancer and breast cancer, respectively. Lung cancer and breast cancer are the two most common origins of BMs [19, 20]. The differentiation of pathological types of primary lung and breast are of critical value in the management of BMs [7, 21].

Although, histopathological examination is still the standard for diagnosis of brain tumors [22], the procedure-related complications of biopsy [23], the heterogeneity, and biological diversity of brain tumors had promoted noninvasive differentiation methods with imaging and radiomics [24]. Radiomics has been widely applied in neurooncology to improve the understanding of the biology and treatment in brain tumors by extracting quantitative features from clinical imaging arrays [25]. Kniep et al. used MRI radiomics and achieved an AUC of 0.64 and 0.82 in the prediction of BM originated from NSCLC and melanoma, respectively [10]. Only the machine learning method of the random forest was used for model construction in previous studies. In this study, the LR model and SVM model were used and a best AUC of 0.717 was achieved with MRI in the differentiation BM from NSCLC and breast cancer. Studies indicated that 3D MRI texture features may improve the differentiation accuracy in some situation with a highest AUC of 0.963 ± 0.054 and 0.947 ± 0.067 were achieved in the differentiation BMs originated from lung cancer and melanoma, and lung cancer and breast cancer, respectively, but the differentiation BM from melanoma and breast cancer was unsuccessful (AUC = 0.607 ± 0.180) [11, 12].

Multimodality imaging has been frequently applied in the clinics to overcome the limitations of the independent techniques. Multimodal radiomics had also been investigated to add value in the diagnosis and treatment evaluation [26, 27]. In one of our previous study, we found that CT radiomics was able to differentiate the primary adenocarcinoma (AD) and squamous cell carcinoma (SCC) for BM patients originated from NSCLC with a highest AUC of 0.828 [28]. In this study, a similar AUC of 0.763 was achieved with CT images in the differentiating BMs from lung cancer and breast cancer. As we can see from the results of this study, the combined CT and MRI radiomics greatly improved the model performance in the differentiating BMs from lung cancer and breast cancer (best AUC = 0.805).

One limitation of this study is that the image data are from a single center. A large sample from multiple centers is needed to further improve the generalizability and stability of our models. Another limitation is that only BMs originated from lung cancer and breast cancer were analyzed in this study, BMs from melanoma, colorectal cancer, and other origins were excluded due to the limited cases available. Moreover, although a highest AUC of 0.805 was achieved in the final testing dataset with the combined model, the lower bound of the AUC in the testing dataset is not ideal, which indicated further validation is needed to confirm model stability. To our knowledge, this study is a first attempt to differentiate BMs according to its primary site of origin based on CT texture features. Multimodal radiomics by integrating more imaging modalities, such as PET and MRI, should be considered in our future studies.

In conclusion, radiomics based on both CT features and MRI features are feasible to differentiate the primary site of origin for BM patients. The combination of CT and MRI radiomics improved the performance in the differentiation BMs originated from lung cancer and breast cancer.

## Figures and Tables

**Table 1:** Demographic and clinical characteristic of the training and testing datasets according to individual tumors.

Characteristics	Training dataset (*N* = 125)		Testing dataset (*N* = 54)		*P* value
Primary site	Lung (*N* = 64)	Breast (*N* = 61)	Lung (*N* = 31)	Breast (*N* = 23)	
Gender, no (%)					0.77
Male	46 (71.9%)	2 (3.3%)	22 (71.0%)	0 (0.0%)	
Female	18 (28.1%)	59 (96.7%)	9 (29.0%)	23 (100.0%)	
Age, mean ± SD (years)	63.3 ± 8.7	47.7 ± 11.1	62.4 ± 1.5	49.2 ± 2.1	0.63
Median age (years)	62.5 (46-79)	46.0 (33-77)	62.0 (46-78)	49.0 (33-74)	

**Table 2:** List of selected radiomic features from CT and MRI images.

Image modality	Filter	Features	*P* value
CT	Original	firstorder_10Percentile	0.005
	Original	glszm_LowGrayLevelZoneEmphasis	0.016
	Log-sigma-2-0-mm	glrlm_ShortRunEmphasis	0.007
	Log-sigma-3-0-mm	glrlm_ShortRunEmphasis	0.007
	Wavelet-HHL	firstorder_90Percentile	0.034
	Wavelet-HHL	glcm_Imc2	0.006
	Wavelet-HLH	glcm_Contrast	0.008
	Wavelet-LHH	glcm_Contrast	0.003
	Wavelet-LHH	glszm_SizeZoneNonUniformityNormalized	0.029
	Wavelet-LLL	glszm_GrayLevelNonUniformityNormalized	0.018

MRI	Log-sigma-4-0-mm	firstorder_Kurtosis	0.024
	Log-sigma-4-0-mm	glszm_SizeZoneNonUniformityNormalized	0.027
	Log-sigma-5-0-mm	glszm_LargeAreaHighGrayLevelEmphasis	0.032
	Wavelet-HHL	glcm_DifferenceAverage	0.009
	Wavelet-HLH	glszm_SizeZoneNonUniformityNormalized	0.036
	Wavelet-HLL	firstorder_InterquartileRange	0.014

**Table 3:** Model performance comparison between models in testing dataset.

Models	CT model		MRI model		Combined model	
	Logistic	SVM	Logistic	SVM	Logistic	SVM
AUC	0.708	0.763	0.715	0.717	0.771	0.805
95% CI	0.571-0.846	0.633-0.893	0.573-0.858	0.574-0.859	0.647-0.896	0.683-0.927
Sensibility	0.452	0.710	0.677	0.806	0.548	0.742
Specificity	0.957	0.826	0.826	0.609	0.957	0.870

AUC: area under curve; SVM: support vector machine.

## Data Availability

The analyzed datasets generated during the study are available from the corresponding authors upon reasonable request.

## Supplementary Materials

Supplementary Materials (1) The extraction parameters for the radiomic features of CT images. (2) The extraction parameters for the radiomic features of MRI images. (3) Table s1.
