# Supplementary material for: Differentiating Primary Tumors for Brain Metastasis with Integrated Radiomics from Multiple Imaging Modalities
Source: Dis Markers. 2022 Sep 26;2022:5147085. doi: 10.1155/2022/5147085 (PMC9529469; doi:10.1155/2022/5147085)
Supplement: Supplementary Materials — (1) The extraction parameters for the radiomic features of CT images. (2) The extraction parameters for the radiomic features of MRI images. (3) Table s1. [file 5147085.f1.docx]

**Supplementary Material**

**1. The extraction parameters for the radiomics features of CT images.**

**2. The extraction parameters for the radiomics features of MR images.**

**3. Table s1.**

**1. The extraction parameters for the radiomics features of CT images.**

CT setting：

imageType:

Original: {}

LoG:

sigma: [1.0, 2.0, 3.0, 4.0, 5.0]

Wavelet: {}

featureClass:

shape:

firstorder:

glcm:

- 'Autocorrelation'

- 'JointAverage'

- 'ClusterProminence'

- 'ClusterShade'

- 'ClusterTendency'

- 'Contrast'

- 'Correlation'

- 'DifferenceAverage'

- 'DifferenceEntropy'

- 'DifferenceVariance'

- 'JointEnergy'

- 'JointEntropy'

- 'Imc1'

- 'Imc2'

- 'Idm'

- 'Idmn'

- 'Id'

- 'Idn'

- 'InverseVariance'

- 'MaximumProbability'

- 'SumEntropy'

- 'SumSquares'

glrlm:

glszm:

gldm:

ngtdm:

setting:

# Normalization:

#normalize: true

#normalizeScale: 500 # This allows you to use more or less the same bin width.

# Resampling:

interpolator: 'sitkBSpline'

resampledPixelSpacing: [1, 1, 1]

padDistance: 10 # Extra padding for large sigma valued LoG filtered images

# Mask validation:

#minimumROIDimensions: 2

#minimumROISize: 50

# Image discretization:

binWidth: 25

# first order specific settings:

voxelArrayShift: 1000

# Misc:

label: 1

**2. The extraction parameters for the radiomics features of MR images.**

MR setting:

imageType:

Original: {}

LoG:

sigma: [2.0, 3.0, 4.0, 5.0]

Wavelet: {}

featureClass:

shape:

firstorder:

glcm:

- 'Autocorrelation'

- 'JointAverage'

- 'ClusterProminence'

- 'ClusterShade'

- 'ClusterTendency'

- 'Contrast'

- 'Correlation'

- 'DifferenceAverage'

- 'DifferenceEntropy'

- 'DifferenceVariance'

- 'JointEnergy'

- 'JointEntropy'

- 'Imc1'

- 'Imc2'

- 'Idm'

- 'Idmn'

- 'Id'

- 'Idn'

- 'InverseVariance'

- 'MaximumProbability'

- 'SumEntropy'

- 'SumSquares'

glrlm:

glszm:

gldm:

ngtdm:

setting:

# Normalization:

normalize: true

normalizeScale: 100 # This allows you to use more or less the same bin width.

# Resampling:

interpolator: 'sitkBSpline'

resampledPixelSpacing: [2, 2, 2]

# Mask validation:

#minimumROIDimensions: 2

#minimumROISize: 50

# Image discretization:

binWidth: 5

# first order specific settings:

voxelArrayShift: 300

# Misc:

label: 1

**3. Table s1.**

Table s1 Demographic and clinical characteristics of the patients

| Characteristics of patients | Primary cancer type | |  |
| --- | --- | --- | --- |
|  | Lung cancer | [mammary cancer](javascript:;) | Total  (N=78) |
|  | (N=53) | (N=25) |  |
| Sex, number (%) |  |  |  |
| male | 38(71.7%) | 1(4%) | 39(50%) |
| female | 15(28.3%) | 24(96%) | 39(50%) |
| Age, mean±SD, years | 62.34±7.82 | 50.80±11.30 | 58.64±10.51 |
| <60 | 18(40.0%) | 19(76%) | 37(47.4%) |
| ≥60 | 35(66.0%) | 6(24%) | 41(52.6%) |
| Median age (years) | 62(46-79) | 52(33-77) | 61(33-79) |
| Number of LNM (average) | 95(1.8) | 84(3.4) | 179(2.3) |

N represents the number of patients
